# Impact of Health Informatics Analyst Education on Job Role, Career Transition, and Skill Development: Survey Study

**DOI:** 10.2196/54427

**Published:** 2024-09-25

**Authors:** Kye Hwa Lee, Jae Ho Lee, Yura Lee, Hyunna Lee, Ji Sung Lee, Hye Jeon Jang, Kun Hee Lee, Jeong Hyun Han, SuJung Jang

**Affiliations:** 1 Department of Information Medicine, Asan Medical Center, Seoul, Republic of Korea; 2 Department of Biomedical Informatics, University of Ulsan College of Medicine, Seoul, Republic of Korea; 3 Department of Emergency Medicine, University of Ulsan College of Medicine, Seoul, Republic of Korea; 4 Big Data Research Center, Asan Institute for Life Sciences, Asan Medical Center, Seoul, Republic of Korea; 5 Clinical Research Center, Asan Institute for Life Sciences, Asan Medical Center, Seoul, Republic of Korea; 6 Department of Biomedical Engineering, Asan Medical Institute of Convergence Science and Technology, University of Ulsan College of Medicine, Seoul, Republic of Korea

**Keywords:** health informatics, health informatics training, informatics training, professional development, training program, digital health technology, informatics workforce, informatics competencies, competencies, job skills, continuing education, data science

## Abstract

**Background:**

Professionals with expertise in health informatics play a crucial role in the digital health sector. Despite efforts to train experts in this field, the specific impact of such training, especially for individuals from diverse academic backgrounds, remains undetermined.

**Objective:**

This study therefore aims to evaluate the effectiveness of an intensive health informatics training program on graduates with respect to their job roles, transitions, and competencies and to provide insights for curriculum design and future research.

**Methods:**

A survey was conducted among 206 students who completed the Advanced Health Informatics Analyst program between 2018 and 2022. The questionnaire comprised four categories: (1) general information about the respondent, (2) changes before and after program completion, (3) the impact of the program on professional practice, and (4) continuing education requirements.

**Results:**

The study received 161 (78.2%) responses from the 206 students. Graduates of the program had diverse academic backgrounds and consequently undertook various informatics tasks after their training. Most graduates (117/161, 72.7%) are now involved in tasks such as data preprocessing, visualizing results for better understanding, and report writing for data processing and analysis. Program participation significantly improved job performance (*P*=.03), especially for those with a master’s degree or higher (odds ratio 2.74, 95% CI 1.08‐6.95) and those from regions other than Seoul or Gyeonggi-do (odds ratio 10.95, 95% CI 1.08‐6.95). A substantial number of respondents indicated that the training had a substantial influence on their career transitions, primarily by providing a better understanding of job roles and generating intrinsic interest in the field.

**Conclusions:**

The integrated practical education program was effective in addressing the diverse needs of trainees from various fields, enhancing their capabilities, and preparing them for the evolving industry demands. This study emphasizes the value of providing specialized training in health informatics for graduates regardless of their discipline.

## Introduction

The field of digital health is rapidly advancing, driven by innovative technologies such as artificial intelligence, big data analytics, digital therapeutics, and embedded medical systems [1]. Amid this dynamic progression, there is a pressing need to cultivate aptitudes and insights to remain abreast of these changes. The growing demand for digital health professionals is evident in the United Kingdom, where almost 90% of health care professionals are projected to require digital proficiencies within the next 2 decades [2]. Additionally, the World Health Organization and World Bank estimate that by 2030, approximately 40 million new health and social care jobs will be created, many of which will be within the field of digital health [3]. Despite the high demand for digital health professionals, there is a substantial gap between the skills health informatics (HI) graduates possess upon graduation and those desired by employers [4,5]. As the health care paradigm shifts toward digitalization, there is an escalating demand for adept professionals capable of conceptualizing, instituting, and overseeing digital health interventions [6]. Current HI educational frameworks, however, fall short of equipping students with the requisite practical acumen [7], leaving many underprepared for the challenges of the profession.

Globally, substantial progress has been made in developing curricula dedicated to HI, with the United States being at the forefront of these efforts. The American Medical Informatics Association saw the need for expertise beyond traditional academic pathways and established an education committee in 2002 [8], leading to the development of the 10×10 Program, a bold initiative to train 10,000 HI specialists from 2005 to 2010. This 10-week program, covering 10 core topics, was designed to cater to a wide range of professionals, from health information managers to system developers. However, the multifaceted nature of HI challenges the feasibility of a one-size-fits-all curriculum [9]. A substantial proportion of professionals work without formal training and have distinct requirements depending on their role and academic background. In response, the International Medical Informatics Association has created a curriculum that aims to deliver tailored professional education to a diverse cohort [10]. However, despite these notable advances, a consistent strategy for an ideal curriculum or training approach for health care professionals of diverse backgrounds—cultural and educational—wishing to explore HI is lacking.

In recent years, clinical informatics has been incorporated into the primary curriculum of medical and graduate schools in South Korea. However, these trainings also remain somewhat limited. Acknowledging the interdisciplinary essence of HI, the South Korean government initiated multiple intermediate informatics education programs. In 2018, a large-scale educational program was launched in collaboration with 3 major universities, offering the Genomic Specialist Training Program, Advanced Health Informatics Analyst (AHIA) Training Program, and Precision Medicine Workforce Training Program. This 5-year program was taught independently of regular university and graduate courses and was offered free of charge to all participants. The AHIA program, in particular, was important owing to its focus on data analysis using real-world hospital data, a skill essential for medical information analysis experts. To cultivate an HI expert within a postgraduate program, a commitment of at least 60 credits or a year of full-time study is mandatory. This bespoke program entailed an annual workload of 56.5 study hours and mirrored the rigor of a postgraduate curriculum. By March 2023, a total of 206 participants from various academic backgrounds and degree pursuits, some with prior experience in relevant fields, had completed the AHIA program. Given the diverse academic trajectories of the attendees and the in-depth instruction in HI, scrutinizing their posttraining professional applications or shifts in employment status was anticipated to yield intriguing insights. This study was therefore conducted to survey the program alumni, aiming to discern the educational impact and gather valuable perspectives to shape subsequent informatics curricula. The overarching objective of this research is to validate the efficacy of intensive HI education when delivered to individuals across a spectrum of academic and professional backgrounds. Such insights will underpin the development and refinement of future informatics curricula.

## Methods

### AHIA Course

The survey was conducted among the 206 students who completed the AHIA course over a period of 5 years (2018‐2022). This advanced course was conducted once a year in 2018 and 2019, and twice a year from 2020 to 2022. On average, each course had 27.4 students enrolled. Each course consisted of 10 sessions. A total of 206 (97.2%) out of 212 students completed the course, and the failure and dropout rate was 2.8% (6/212). The program is designed to provide practice-oriented education, allowing students to experience the practical needs and challenges faced in the medical field. The curriculum encompasses a range of topics, including electronic medical records, medical images, public health, lifelog data, biosignals, and genome data, which are essential in the medical field. A lifelog is a practice where individuals digitally document their daily experiences with different levels of granularity, serving various aims [11]. Theoretical knowledge and practical training were incorporated into the course, which included a 3-week team project to practice problem-solving skills relevant to real-world scenarios. The curriculum and structure of this course are provided in Multimedia Appendix 1.

### Survey Design

We implemented a structured survey to systematically evaluate and analyze the insights for future informatics curriculum design and assess the effectiveness of our training approaches. The questionnaire was organized into four categories, as outlined in Table 1: (1) general information on the respondents: this section collected essential demographic data about the survey respondents; (2) changes before and after program completion: participants were asked to reflect on any changes they experienced in their knowledge or skills after completing the professional program; (3) impact of the professional program on professional practice: this section aimed to assess how the program influenced the professional practices of the participants; and (4) continuing education requirements: this section sought to identify any specific needs or preferences for further education among the respondents. The questionnaire incorporated adaptive questioning techniques to minimize the quantity and complexity of the questions presented. The number of questionnaire items per page was from 2 to 4. The questionnaire was distributed over 12 pages. The respondent was able to modify their response by using the back button. The questionnaire is provided in Multimedia Appendix 2.

**Table 1.** Questionnaire composition and questions.

Category	Classification	Questions, n
General information	Sex Age Residence Final education Major Employment status Occupation Organization Job before and after completing the program Duration of work experience Work related to informatics or health informatics	12
Changes before and after completing the intensive course	Purpose or reason for program application Current work impact Changes in work after completing the program How helpful (satisfied) was the course? Positive impact A change in career or intention to change careers in informatics or health informatics after completing the program	8
Effects of advanced courses on informatics in performing tasks	Level of change in medical data analysis and processing ability Performed tasks related to informatics or health informatics Change in fear of working with informatics or health informatics Participation in informatics- or health informatics–related activities Improved skills and abilities related to informatics or health informatics Change in interest in informatics or health informatics Change in participation in informatics- or health informatics–related activities Reasons why informatics- or health informatics–related activities were difficult to do	6
Demand for continuing education	Continuing education or reinforcement after completing the course Demand for informatics- or health informatics–related personnel in your organization Cultivation of specialized personnel related to informatics or health informatics Improvements and suggestions	5

### Evaluation of the Reliability and Validity of the Survey

The survey was designed to align with the educational objectives and learning outcomes of the AHIA course. The purpose was to evaluate the applicability of the theoretical knowledge and practical training in addressing real-world health care challenges. Survey questions were developed to assess shifts in students’ knowledge and skills, the impact on their professional practices, and their needs for continuing education. The initial draft of the survey was designed by our research team of HI experts to ensure the relevance and clarity of the questions, thereby enhancing the survey’s reliability and validity. To ascertain the reliability and validity of the survey, we engaged a panel of experts in HI to review and critique the initial survey draft. Their invaluable feedback led to the refinement of our survey questions, ensuring they effectively captured the educational outcomes and experiences of our students. This step was crucial in validating the survey instrument and ensuring that the data collected were both reliable and reflective of the course’s impact.

### Participant Recruitment and Data Collection

From January 30 to February 8, 2023, data were gathered using a web-based questionnaire. Links to this questionnaire were emailed to the 206 students who had completed the AHIA course between 2018 and 2022. The first page of the survey provided information on the purpose of the research, the length of time needed to complete the survey, and the methods of personal information protection.

### Statistical Analysis

The collected survey data underwent a comprehensive statistical analysis. Only completed survey data were analyzed. First, basic descriptive analysis techniques were applied to examine the characteristics of the survey respondents, including computing measures such as mean, median, and SD to summarize the central tendency, dispersion, and distribution of the data. Additionally, frequency tables were used to present categorical variables, providing insights into the demographic composition of the respondents. Furthermore, ANOVA was conducted to determine if there were significant differences in informatics medicine work based on the major of the education graduates. Visualization methods, such as pie graphs, were used to illustrate differences among majors and to depict shifts in proficiency levels before and after the training course. All statistical analyses were performed using R software (version 4.1.1; R Foundation for Statistical Computing).

### Ethical Considerations

This research paper was reviewed and approved by the Institutional Review Board of Asan Medical Center (S2022-2671-0001). Before commencing the survey, participants were mandated to provide their informed consent, acknowledging the study’s objectives and permitting the collection of their personal data. Participation was voluntary, and upon completing all items in the survey, participants were compensated with a small reward. The collected responses were anonymous and used exclusively for analysis and deriving outcomes, with the confidentiality of individual responses being strictly protected under Article 33 of the Statistics Act.

## Results

### Participant Demographics

Among the 206 trainees, 161 (78.2%) responded. Due to the anonymous nature of the survey, identifying the nonrespondents was not feasible, and in comparison to prior research [8], achieving a response rate of 78.2% is considered substantial. This sample size was deemed sufficient to draw meaningful conclusions, with the sampling error, expressed as a margin of error, calculated to be ±3.62% at a 95% confidence level. Table 2 presents the demographic characteristics of the study population. The sex distribution shows that of the 161 respondents, 54% (n=87) were female and 46% (n=74) were male, with no statistically significant difference between the 2 groups (*P*=.31). The age distribution was as follows: 52.9% (46/87) of female participants and 35.1% (26/74) of male participants were in the 20‐29 years group; 28.7% (25/87) of female participants and 37.9% (28/74) of male participants were in the 30‐39 years group; and 18.4% (16/87) of female participants and 27% (20/74) of male participants were in the 40+ years group. No significant difference between the age of male and female participants was observed (*P*=.08). There were also no significant educational differences between the sexes (*P*=.25). Most participants had a bachelor’s degree (34/87, 39.1% of female participants and 19/74, 25.7% of male participants) or a master’s degree (39/87, 44.8% of female participants and 38/74, 51.4% of male participants), while a smaller proportion had a high school diploma or a PhD. Job status differed significantly between female and male participants (*P*=.01). Most female participants (52/87, 59.8%) and male participants (56/74, 64.4%) were employed in full-time jobs, while smaller proportions were engaged in job preparation or postgraduate studies. Lastly, there were no significant differences in the locations between the sexes (*P*=.14). Most participants (62/87, 71.3% of female participants and 42/74, 48.3% of male participants) were from Seoul, followed by Gyeonggi-do and other regions.

**Figure 1.**
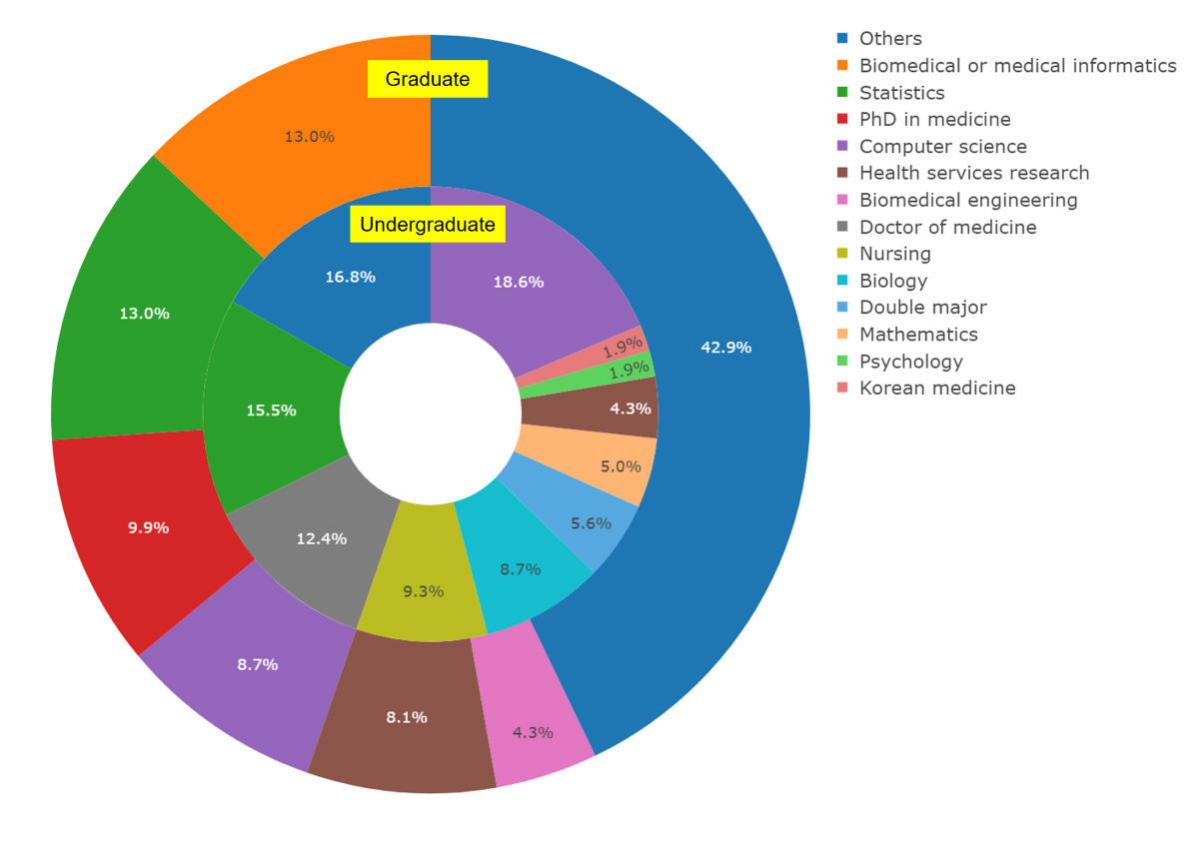
Respondents’ undergraduate and graduate majors.

Among the 161 respondents, 64% (n=103) had a master’s degree, PhD, or higher. These respondents’ undergraduate majors and changes in their majors in the master’s and doctoral courses after the bachelor’s degree were compared. The most common undergraduate majors were computer science (n=30, 18.6%), “others” (n=26, 16.1%), statistics (n=25, 15.5%), medicine (n=20, 12.4%), and nursing (n=15, 9.3%), whereas for the master’s degree or doctoral major, “others” was the most common at 42.9% (n=69), followed by biomedical or medical informatics and statistics at 13% (n=21) each, medicine at 9.9% (n=16), computer science at 8.7% (n=14), health service research at 8.1% (n=13), and biomedical engineering at 4.3% (n=7). “Others” includes experimental psychology, molecular science, microbiology, psychology, etc (Figure 1).

The primary motivation for enrolling in the AHIA course and the reason for applying to informatics-related jobs were examined. The highest proportion of respondents (n=120, 74.5%) indicated that they sought to enhance or improve their job competencies, followed by 67.1% (n=108) who applied to enhance their future job prospects. Most respondents (n=120, 74.5%) joined the course to strengthen their job-related skills and performance. Moreover, a substantial proportion (n=70, 43.5%) expressed their interest in learning new technologies with promising opportunities, 36% (n=58) highlighted interest in joint research or collaboration, and 32.3% (n=52) considered leveraging the potential for collaboration, indicating the high expectations for collaboration and growth opportunities in the converging field. Furthermore, some participants (n=47, 29.2%) stated that they pursued the course to obtain a completion certificate, and 6.8% (n=11) indicated that they received recommendations or endorsements from others as motivation for enrollment.

**Table 2.** Respondent demographics.

Variables		Female sex (n=87)	Male sex (n=74)	*P* value
Respondents (n=161), n (%)		87 (54)	74 (46)	.31
**Age group (years), n (%)**				.08
	20‐29	46 (52.9)	26 (35.1)	
	30‐39	25 (28.7)	28 (37.9)	
	40+	16 (18.4)	20 (27)	
**Education, n (%)**				.25
	High school diploma	3 (3.4)	2 (2.7)	
	Bachelor’s degree	34 (39.1)	19 (25.7)	
	Master’s degree	39 (44.8)	38 (51.4)	
	PhD	11 (12.6)	15 (20.3)	
**Job status, n (%)**				.01
	Job preparation	13 (14.9)	1 (1.1)	
	Postgraduate student	15 (17.2)	11 (12.6)	
	Full-time job	52 (59.8)	56 (64.4)	
	Part-time job	5 (5.7)	6 (6.9)	
**Region, n (%)**				.14
	Seoul	62 (71.3)	42 (48.3)	
	Gyeonggi-do	17 (19.5)	20 (23)	
	Other	8 (9.2)	12 (13.8)	

### Informatics Ability After the Training Course

Regarding the current medicine-related work of respondents according to their majors, 117 (72.7%) of the 161 respondents answered that they “preprocess the collected data”; 57.1% (n=92) noted that they “visualize the results to help understand the main analysis results”; and 57.1% (n=92) said that they “write an analysis report applying various modeling techniques.” To identify a difference in the information medicine work currently performed according to the final major of the education graduates, data were visualized according to majors (ANOVA; *P*<.001). With the exception of graduates with mathematics majors, most graduates (n=117, 72.7%) performed plenty of data preprocessing (Multimedia Appendix 3). Data preprocessing was identified as the main task for graduates of health science research, statistics, and computer science majors. Graduates of medical (doctors and nurses) and double majors often performed tasks related to data utilization plan establishment, and data analysis was performed frequently by graduates with mathematics, statistics, and “others” majors.

### Changes in Technology and Knowledge After the Training Course

**Figure 2.**
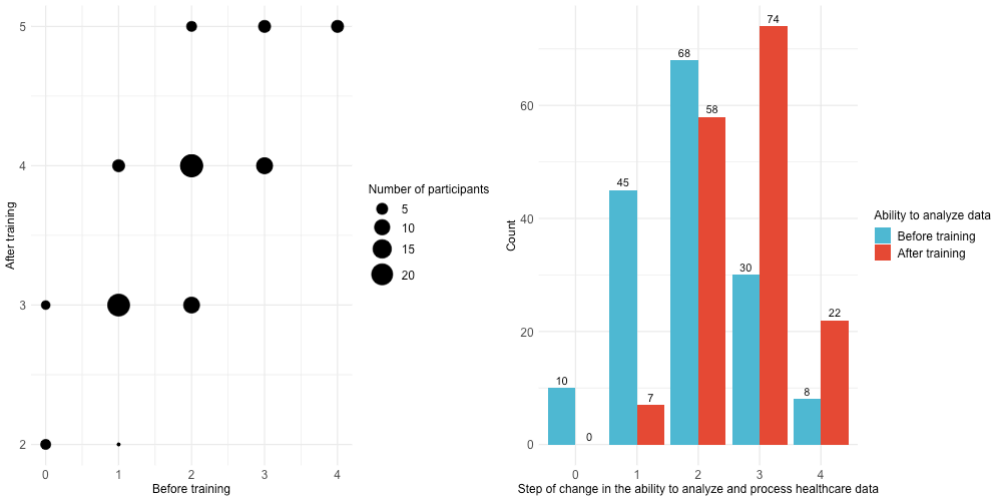
Step of change in medical data analysis and processing capabilities. Steps: 0=I did not know at all, 1=I understand the concept after hearing the term, 2=I can see and follow the analysis method, 3=I know and can design the analysis method, and 4=Expert-level analysis and results can be drawn.

Regarding the ability to analyze and process medical data before and after the training course, most (68/161, 42.2%) respondents selected “I can see and follow the analysis method (Step 2)” before the course. This stage primarily involves the ability to visually recognize and replicate given analysis methods based on instructional guidance, which is crucial for foundational learning and initial engagement with medical data analysis. However, after completing the course, 46% (n=74) said “I know and can design the analysis method (Step 3),” indicating that medical data analysis skills and processing abilities had improved upon completion. This progression signifies a deeper understanding and ability, not just to follow but also to design and conceptualize analysis methods independently. Step 3 encompasses a critical transition from merely executing predefined analysis steps to creating customized analysis frameworks suited to specific medical data challenges. Additionally, of the 6.2% (n=10) of respondents who answered “I did not know at all (Step 0)” before completion, half (5/10, 50%) of them improved to “I understand the concept after hearing the term (Step 1),” while the other half (5/10, 50%) moved to “I can see and follow the analysis method (Step 2),” highlighting the improvement from the beginner to the intermediate step (Figure 2 and Table 3). After completion, 111 (68.9%) students improved their skills by at least 1 step. Excluding the 8 (5%) individuals who began the course at the fifth step, 67.3% (103/153) showed a technical improvement of at least 1 step.

**Table 3.** Step of change in medical data analysis and processing capabilities (n=161).

Step	Degree of change	Before training, n (%)	After training, n (%)
0	I did not know at all	10 (6.2)	0 (0)
1	I understand the concept after hearing the term	45 (28)	7 (4.3)
2	I can see and follow the analysis method	68 (42.2)	58 (36)
3	I know and can design the analysis method	30 (18.6)	74 (46)
4	Expert-level analysis and results can be drawn	8 (5)	22 (13.7)

### Shift in Job Change Intentions Before and After Training

After completion of the AHIA course, 58.4% (94/161) of respondents in the field of informatics or HI changed jobs or intended to change jobs. Although more than half expressed this intent, actual job change was relatively rare, with only 22.4% (36/161) experiencing a job change. Among those who responded to further questions (n=91), except for 3 who did not respond, 82.4% (n=75) stated that the course influenced their decision. Specifically, “understanding work contents and characteristics in the field of informatics” (n=58, 63.7%) and “interest in the informatics field” (n=56, 61.5%) were cited as significant factors influencing either the experience of a job change or the intention to change jobs (as depicted in Table 4).

**Table 4.** Impact of the Advanced Health Informatics Analyst course on job change or the intention to change jobs.

Factors that impacted job change or the intention to change jobs	Respondents (n=91; 91/161, 56.5%)
Understanding work contents and characteristics in the field of informatics	58 (63.7)
Interest in the informatics field	56 (61.5)
Confirmation of the potential for growth in the area of IT	50 (54.9)
Improving individual academic skills and gaining academic qualifications	42 (46.2)
Developing jobs skills (like developing technology, analyzing data, etc)	38 (41.8)
Confirmation that the area of informatics and the person’s skills are a good match	36 (39)
Exchange with people interested in medical information	30 (33)

### Impact of the AHIA Course on Informatics Activities and Attitudes

To assess the influence of the AHIA course on activities and attitudes within the field of informatics, we formulated 4 questions. These were rated on a Likert scale across 3 key areas: the type of positive impact the course had, the increase in activities related to informatics, and changes in interest toward or apprehension about the field of informatics. Among the 161 respondents, 72.6% (n=117) answered that the course had a positive effect. Among them, 75.2% (n=121) and 60.2% (n=97) highlighted that they had “enhanced knowledge in informatics for personal improvement” and “gained practical experience in utilizing data for academic or professional purposes,” respectively. Regarding the increase in activities in the informatics field and changes in attitudes, 63.4% (n=102) of the respondents stated that their participation in informatics activities had increased. Additionally, 90.1% (n=145) of respondents reported that their interest in informatics increased and 73.9% (n=119) said their fear had decreased.

### Examining the Link Between the Impact of the AHIA Course and Respondent Characteristics

To confirm the relationship between the influence of the AHIA course and the characteristics of the respondents, we performed a multivariable logistic analysis for each outcome by adding the age, sex, education level, and field of work of the respondents. Participants showing a positive correlation in the effect of the AHIA course on job performance were those with a master’s degree, PhD, or higher (odds ratio [OR] 2.74, 95% CI 1.08‐6.95) and workers in regions other than Seoul and Gyeonggi-do (OR 10.95, 95% CI 1.08‐6.95). The respondent factor significantly associated with increased informatics activity was having a master’s degree or higher (OR 2.84, 95% CI 1.27‐6.31; *P*=.01), while other factors did not show any correlation. Regarding the increase in informatics activities, the highest observed proportion (83/161, 51.6%) involved the “use of informatics in actual work,” followed by “completing an additional degree in informatics” at 26.7% (43/161), suggesting that these degree-related matters are closely related factors. Respondent sex was associated with increased interest in informatics, and female participants responded that they were more interested than male participants (OR 3.51, 95% CI 1.10‐11.21). Age showed no significant difference in all 4 items (Table 5).

**Table 5.** Course effects on health informatics work and attitudes.

Variables		Effect			Activity			Interest			Fear decreased		
		OR^a^ (95% CI)		*P* value	OR (95% CI)		*P* value	OR (95% CI)		*P* value	OR (95% CI)		*P* value
**Age group (years; reference: 20-29)**													
	30‐39	0.43 (0.16-1.14)		.009	0.78 (0.33-1.84)		.57	0.88 (0.19-4.08)		.87	0.77 (0.31-1.94)		.58
	40+	0.98 (0.31-3.08)		.10	1.00 (0.38-2.65)		.99	0.63 (0.14-2.93)		.56	0.92 (0.33-2.58)		.88
**Sex (reference: male)**													
	Female	1.59 (0.75-3.36)		.23	1.55 (0.78-3.08)		.21	3.51 (1.10-11.21)		.03	1.40 (0.67-2.91)		.37
**Education (reference: bachelor’s degree)**													
	Master’s degree or PhD	2.74 (1.08-6.95)		.03	2.84 (1.27-6.31)		.01	3.69 (0.96-14.23)		.06	1.30 (0.55-3.07)		.55
**Location (reference: Seoul)**													
	Gyeonggi-do	1.22 (0.51-2.89)		.66	0.96 (0.43-2.15)		.92	1.07 (0.30-3.90)		.91	1.11 (0.46-2.69)		.82
	Other areas	10.95 (1.34-89.33)		.02	0.73 (0.26-1.99)		.53	1.30 (0.25-6.85)		.76	0.66 (0.24-1.87)		.44

a OR: odds ratio.

## Discussion

Our study investigated the changes observed in graduates who completed the 56-hour medical information specialist education course, which was offered across a period of 5 years. Graduates from this course had diverse academic backgrounds, and their postcourse informatics work varied accordingly. Graduates of health sciences, statistics, and computer engineering majors primarily focused on data preprocessing. Medical professionals and those with double majors often worked on data utilization plans, while those from mathematics and statistics backgrounds frequently engaged in data analysis. The faculty involved in delivering the course, comprising professors from multiple universities including some authors of this paper, primarily aimed to enhance job competency or equip participants with the necessary skills for future employment opportunities. Regarding the informatics work currently performed by the graduates, most (116/161, 72.2%) cited tasks such as “preprocessing the collected data,” “visualizing the results to understand the main analysis outcomes,” and “processing the data and preparing an analysis report using various modeling techniques.” Additionally, local workers with a master’s degree or higher reported experiencing the greatest positive impact from the course. A significant proportion of graduates also indicated that their education impacted their career transitions. Predominantly, comprehension of job roles and characteristics, along with an intrinsic interest in the field, emerged as the primary influencing factors. The results indicate that this convergence practice education program serves as a successful model, effectively addressing the needs of trainees from various fields, enhancing their competencies, and preparing them for the evolving demands of the industry.

This study demonstrates the value of using real-world clinical data for training individuals across various academic fields within HI. The finding that 63.4% (102/161) of participants became more engaged in informatics-related endeavors after the course is compelling evidence for the effectiveness of this educational method in enhancing individual proficiency and involvement. Furthermore, our data suggest that hands-on clinical data education benefits those from diverse academic backgrounds, augmenting their HI activities. This aligns with prior research underscoring the value of hands-on experience in the competencies desired in industry professionals [12]. This could be an important factor to consider in future educational curriculum development or professional training programs. Interestingly, respondents holding a master’s degree or higher, or those working in local settings, indicated the most significant benefits from the program. These findings imply that individuals with higher degrees possess a solid grounding in foundational HI concepts. While industry hiring trends often favor individuals with a bachelor’s degree [12,13], those with advanced degrees are positioned to derive maximum value from HI training. Their robust foundation in core HI areas, including computer science, statistics, and health sciences, enables them to fully grasp and effectively implement HI concepts. In a field such as HI, wherein cultivating interdisciplinary expertise is crucial, offering specialized courses rooted in real data to master’s-level professionals from varied backgrounds can be a potent strategy for developing top-tier talent. Notably, our findings suggest that graduates in rural regions benefited more from the training compared to their counterparts in well-resourced areas such as Seoul or Gyeonggi-do. This highlights the potential of similar targeted programs to elevate the educational standard and bridge the gap in educational opportunities across regions.

In well-resourced nations, medical system computerization is on the rise, with 96% of the general hospitals in South Korea adopting electronic medical records [14]. These health care digital shifts produce vast data volumes that are essential for decision-making, gauging treatment efficacy, and underpinning evidence-based approaches [15]. To efficiently handle such expansive medical data, experts in medical information are imperative at every phase [16]. As the convergence of health care and technology accelerates, academic institutions are striving to cater to varied aspirants, spanning recent high school graduates to IT and medical professionals [17]. However, there is a scarcity of programs tailored for the HI sector, which is vaguely defined [18,19]. A disparity in the provision of HI degrees exists even in well-equipped nations such as the United States [20]. The 10×10 Program by the American Medical Informatics Association stands out as a robust training model [9]; however, for countries lacking ample educational resources, state-backed initiatives resembling the AHIA course offer a potential solution. The International Medical Informatics Association advocates for dedicated establishments offering continuous education courses [10]. Given the dynamic landscape of health IT, future programs must embed lifelong learning tenets, encouraging graduates to persistently upskill. HI professional development should also extend to current medical staff and those from varied academic realms. Training individuals with profound health care and IT expertise, coupled with a hands-on grasp of the health care structure, remains a formidable challenge. The graduates of the AHIA course, with their varied academic histories, highlight the need for more holistic, adaptive future programs. The assertion by Topol [2] regarding the imminent digital skill requirement for all National Health Services roles emphasizes the paramount importance of digital literacy in health care. Despite the focus of the program being professionals and researchers, 6.2% (10/161) of respondents professed ignorance of informatics jargon. This underscores the pivotal role of informatics education in molding a competent health workforce. Moreover, those possessing an advanced degree reportedly reaped the most benefit from this training, suggesting that upcoming courses should be tailored to the unique educational backgrounds of enrollees.

AHIA graduates hail from a broad array of fields, including computer science, health care, and statistics, with a wide range of focus in their advanced studies as well. While HI skills are crucial, medical schools offer minimal, inconsistent, and rarely updated education, leaving students unprepared for the digital health care landscape [21]. Although the swift evolution of medical technology necessitates continual adaptation of research and educational content within the field, it appears that updates to the undergraduate HI curriculum lag behind these technological advancements. Our findings highlight that, in the fast-evolving domain of medical informatics, implementing a robust and comprehensive curriculum that transcends the confines of specific academic departments or institutions—particularly one that caters to the interdisciplinary education of students from a variety of academic backgrounds—emerges as a crucial strategy. This approach is essential for keeping pace with the rapid developments in the field. We also explored the impact of the program on the career paths of graduates, finding that a majority were willing to consider job changes and that a notable portion had already done so. They attributed a significant influence on their career decisions to the program. Analyzing web-based job postings, a German study revealed a high demand for medical informatics professionals, with half of the jobs concentrated in hospitals [22]. While hands-on experience is crucial, employers and graduates alike find it challenging to find programs that bridge the gap between computer science expertise and practical hospital knowledge. Considering that the AHIA program offers diverse students hands-on training and real-world experience using actual hospital data while working on projects in teams, the AHIA model suggests a positive strategy for strengthening the competencies of students entering the HI job market and meeting job demands.

Despite its contributions to understanding the impact of an AHIA course on graduates’ job roles and competencies, this study acknowledges several limitations. First, the survey’s broad focus on the overall educational program might have overlooked the nuanced impacts of specific subjects, such as information security and privacy, on graduates’ career outcomes. While this approach captures the general effectiveness of the program, it leaves room for further exploration into how individual modules shape professional skills and knowledge. Potential biases in survey responses due to their self-reported nature represent another limitation. While measures were taken to ensure anonymity and encourage candidness, the inherent nature of self-reporting might introduce biases that could affect the interpretation of our findings. This aspect underscores the need for caution in generalizing the results beyond the surveyed population. The generalizability of our findings is also limited by the specific context of the educational program and its participants. This specificity might not fully capture the diverse experiences across the broader field of HI, suggesting that the findings might not be universally applicable without further validation in different settings. Furthermore, the data collection process, focusing on immediate postgraduation outcomes, does not account for the longitudinal development of competencies or career progression. This temporal limitation highlights the potential for more comprehensive, longitudinal studies to understand the lasting impacts of educational programs. Lastly, our study could not evaluate the characteristics of nonrespondents. A total of 21.8% (45/206) of individuals who completed the course did not participate in the subsequent survey. While survey fatigue, time constraints, or privacy concerns might have contributed to this nonresponse rate, the specific reasons for their lack of participation remain unexplored due to the anonymized nature of our data collection process. This limitation prevents us from fully understanding whether the nonrespondents possess distinct demographic or professional characteristics compared to survey participants, which could potentially introduce a bias in our findings. Despite these limitations, our research offers significant insights into the value of HI education and its role in preparing professionals for the field. It underscores the importance of curriculum design in addressing the evolving needs of the HI sector and provides a foundation for future research.

Our survey of AHIA graduates revealed 2 particularly compelling findings that enrich our understanding of the program’s effectiveness and potential areas for future research. First, the reported educational impact of the AHIA program was more pronounced among graduates holding a master’s degree or higher. Second, students residing outside the metropolitan area of Seoul perceived a greater benefit from their participation in the program. These results suggest that advanced educational background and geographic diversity play significant roles in the perceived value and impact of HI education. Incorporating these perspectives, we propose additional areas for future research. Further investigation into the influence of prior academic achievements on the outcomes of HI education is warranted. Specifically, understanding the mechanisms through which graduates with higher degrees report greater benefits from their education can inform the tailoring of programs to enhance benefits across various educational levels. This exploration is essential for developing curricula that are responsive to the educational background of students, ensuring that all participants can achieve significant gains from their involvement. Additionally, the greater perceived impact of HI programs among students from nonmetropolitan areas highlights the need for an in-depth analysis of geographic disparities in educational outcomes. Identifying the challenges faced by students in accessing traditional educational resources and the ways in which alternative models such as the AHIA program can effectively bridge these gaps will be crucial. Such research could lead to the development of more accessible and inclusive HI education programs, which are particularly vital for students in resource-limited countries and those residing outside major urban centers.

Timely, project-based, and government-supported education programs similar in rigor to degree programs can provide practical education that helps professionals in HI meet the ever-changing needs of the field and continue to upskill. As a case study of a flexible educational program that can accommodate a variety of educational methods and topics, the experience and results of the AHIA program can act as a reference for the global HI education community in the design of integrated educational modules centered on real data and cases.

In conclusion, this study underscores the necessity of flexible, inclusive, and responsive educational models in HI. By addressing the highlighted areas for future research, the field can move toward developing educational programs that not only cater to the diverse needs of students but also prepare them to meet the challenges and opportunities within the dynamic landscape of HI.

## Supplementary material

10.2196/54427 Multimedia Appendix 1 Advanced Health Informatics Analyst course training modules.

10.2196/54427 Multimedia Appendix 2 Survey on changes in informatics competency and demand for continuing education among graduates after completing the Advanced Health Informatics Analyst course.

10.2196/54427 Multimedia Appendix 3 Current informatics work sectors of respondents according to their majors.

## Abbreviations

AHIA: Advanced Health Informatics Analyst
HI: health informatics
OR: odds ratio
