# Supplementary material for: Impact of Health Informatics Analyst Education on Job Role, Career Transition, and Skill Development: Survey Study
Source: JMIR Med Educ. 2024 Sep 25;10:e54427. doi: 10.2196/54427 (PMC11446175; doi:10.2196/54427)
Supplement: Multimedia Appendix 1 [file mededu-v10-e54427-s001.doc]

**Advanced Health Informatics Analyst Course Training Modules**

The curriculum encompassed foundational lectures on key subjects in health informatics as well as data-centered practices. Additionally, specialized lectures were provided. In the culmination of the course, teams were assembled based on their data specialties and analysis methodologies. These teams were then assessed through the outcomes of their team projects.

- Core Curriculum

| No | Subject | Episodes | Title |
| --- | --- | --- | --- |
| 1 | Introduction to Biomedical Informatics | 1 | Bio-Medical Information Overview |
|  |  | 2 | Future of Medicine and Digital Health |
|  |  | 3 | Role of BMI/Health Information Technology |
|  |  | 4 | Challenges of BMI/HIT Digital Health, Experts in BMI |
| 2 | Characteristics of Medical Data | 5 | Characteristics of medical materials |
|  |  | 6 | Types of Medical Materials |
|  |  | 7 | Precautions for Using Medical Resources |
| 3 | Medical terminology, taxonomy, standardization, ontology | 8 | Medical terminology, taxonomy, standardization, and ontology overview |
|  |  | 9 | The Importance of Healthcare Terminology/Taxonomy/Standards |
|  |  | 10 | Examples of healthcare terminology: ICD, LOINC, SNOMED-CT, ATC, RxNorm... |
|  |  | 11 | Examples of Healthcare Terminology: Nursing Terminology, MeSH... |
|  |  | 12 | Relationship between medical terminology and classification system, status of domestic use |
| 4 | Healthcare Information Systems and Databases | 13 | Overview of the Health Information Database (Required) |
|  |  | 14 | Database Concepts |
|  |  | 15 | Build and use databases |
|  |  | 16 | Database Utilization Prospects |
| 5 | Hospital Information System (EMR/OCS) | 17 | Hospital Information System (HIS) Overview, HIS in Korea |
|  |  | 18 | Order Communication System (OCS), CPOE, CDSS |
|  |  | 19 | Electronic Medical Records (EMR), m-EMR |
|  |  | 20 | Other HIS: PACS, CRDW; Next-Generation EMR |
| 6 | Clinical Decision Support System (CDSS) | 21 | Overview of Clinical Decision-Making System |
|  |  | 22 | History of CDSS |
|  |  | 23 | CDSS for Clinical Practice |
|  |  | 24 | CDSS Domestic Status and Future Prospects |
| 7 | Research Information Systems and Clinical Research Informatics | 25 | Clinical Research Informatics Overview |
|  |  | 26 | Generation, extraction, and processing of data |
|  |  | 27 | EMR Data-Based Analysis Cases |
|  |  | 28 | EMR Data Utilization Research/Industrialization Prospects |
| 8 | Clinical Trials and Data Management | 29 | Introduction to Clinical Trial |
|  |  | 30 | Introduction to Clinical Data Management |
|  |  | 31 | Data Validation |
|  |  | 32 | e-CRF |
| 9 | EMR-based research | 33 | Types and Schemes of Clinical Research |
|  |  | 34 | Retrospective cohort study design |
|  |  | 35 | EMR-based research case study |
| 10 | Medical Imaging with Artificial Intelligence | 36 | Healthcare Artificial Intelligence Overview |
|  |  | 37 | Utilization of Artificial Intelligence in Medical Image Analysis |
|  |  | 38 | Examples of medical image analysis using artificial intelligence |
|  |  | 39 | Future Prospects for Artificial Intelligence Utilization |
| 11 | Genomic Information Analysis and Personalized Medicine | 40 | Genomics and DNA sequencing |
|  |  | 41 | Cancer Genomics, NGS Gene Panel Test |
|  |  | 42 | Details of Data analysis workflow: Raw data～ |
|  |  | 43 | Details of Data analysis workflow: Base quality recalibration～ |
|  |  | 44 | Examples of NGS panel test in clinic |
| 12 | Big Data, Artificial Intelligence, and Cloud in Healthcare | 45 | Healthcare big data overview |
|  |  | 46 | Healthcare big data R&D/Brand new data |
|  |  | 47 | Artificial Intelligence: Terminology, Types of Machine Learning, Data Importance, Current State of Healthcare AI |
|  |  | 48 | Cloud, what's next |
| 13 | Understanding Digital Health | 49 | Digital Health Overview |
|  |  | 50 | Digital Health Domain/Domestic Status |
|  |  | 51 | The Need for Digital Health |
|  |  | 52 | Digital Health Cautions, Future Prospects |
| 14 | Department of Consumer Health Informatics Personal Health Record System | 53 | Customer Health Informatics |
|  |  | 54 | CHI Concepts & Examples |
|  |  | 55 | Personal Health Record (PHR), Examples of PHRs |
|  |  | 56 | Next Generation Mobile PHR |
| 15 | Understanding Information Security and Privacy | 57 | Privacy Protection Cases and Issues |
|  |  | 58 | What is personal information? |
|  |  | 59 | Laws and Regulations on the Protection of Personal Information |
|  |  | 60 | Personal Information Protection Activities Status and Prospects |

- Lectures and advanced practice

- Use cases and in-depth practice of healthcare data analysis

: EMR data, medical image data, vital sign data, public data, life log data

- Team Projects

- Cloud Usage/ Analytics Dataset Coaching

- Subject matter team project exercises: EMR data, medical imaging data, vital sign data, public data

- 50 hours of lectures and practical training

| system | Training Orientation & Graduation Ceremony | Introduction to  Health Informatics | Lectures and Further Practice  (EMR Data) | Lectures and Further Practice  (Video  Data) | Lectures and Further Practice  (Vital Signs  Data) | Lectures and Further Practice  (Open Government  Data) | Particulars  (Life Log data) | team  project |
| --- | --- | --- | --- | --- | --- | --- | --- | --- |
| 50 hours | 10 hours | 8 hours | 5 hours | 5 hours | 5 hours | 5 hours | 4 hours | 8 hours |
|  | 20% | 16% | 10% | 10% | 10% | 10% | 8% | 16% |

◯ Detailed training program for advanced courses

| Week | Time | Lecture Hours | Course Type | Subject | Curriculum |
| --- | --- | --- | --- | --- | --- |
| Week 1 | 13:00∼14:00 | 1 | theory | Orientation | Course Introduction & Orientation |
|  | 14:00∼15:00 | 1 | theory |  | Healthcare Big Data Policy Status and Direction |
|  | 15:00∼16:00 | 1 | theory |  | Future Medicine through Big Data |
|  | 16:00∼17:00 | 1 | theory |  | Clinical Field Needs, Big Data Utilization, and Research Cases |
|  | 17:00∼19:00 | 2 | theory |  | A time of harmony and communication among trainees (dinner) |
| Week 2 | 13:00∼14:00 | 1 | theory | Introduction 1 | Introduction to Biomedical Informatics |
|  | 14:00∼15:00 | 1 | theory |  | Generation and Utilization of Healthcare Data 1 - Video |
|  | 15:00∼16:00 | 1 | theory |  | Generation and Utilization of Healthcare Data 2 - Genome |
|  | 16:00∼17:00 | 1 | theory |  | Creation and Utilization of Healthcare Data 3 - EMR |
| Week 3 | 13:00∼14:00 | 1 | theory | Introduction 2 | Asan Medical Center Health Data Storage and Utilization Status |
|  | 14:00∼15:00 | 1 | theory |  | Generation and Utilization of Healthcare Data 4 - Vital Signs |
|  | 15:00∼16:00 | 1 | theory |  | Creation and Utilization of Healthcare Data 5 - Open Government Data |
|  | 16:00∼17:00 | 1 | theory |  | Creation and Utilization of Healthcare Data 6 - Lifelog data |
| Week 4 | 13:00∼14:30 | 1.5 | theory | Topics & Advanced Practicum 1 | EMR Data Analytics Use Cases - Enterprise |
|  | 14:30∼16:00 | 1.5 | theory |  | Use Cases for EMR Data Analytics - Researcher |
|  | 16:00∼18:00 | 2 | practice |  | EMR Data Practice |
| Week 5 | 13:00∼14:30 | 1.5 | theory | Topics & Advanced Practicum 2 | Use Cases of Medical Image Data Analysis - Companies |
|  | 14:30∼16:00 | 1.5 | theory |  | Use Cases of Medical Image Data Analysis - Researcher |
|  | 16:00∼18:00 | 2 | practice |  | Medical Imaging Data Practice |
| Week 6 | 13:00∼14:30 | 1.5 | theory | Topics and Advanced Practicum 3 | Use Cases of Vital Signs Data Analysis - Companies |
|  | 14:30∼16:00 | 1.5 | theory |  | Use Cases of Vital Signs Data Analysis - Researcher |
|  | 16:00∼18:00 | 2 | practice |  | Intensive Practice of Vital Signs Data |
| Week 7 | 13:00∼14:30 | 1.5 | theory | Topics and Further Practicum 4 | Public Healthcare Big Data Use Cases - Enterprise |
|  | 14:30∼16:00 | 1.5 | theory |  | Public Healthcare Big Data Use Case - Researcher |
|  | 16:00∼18:00 | 2 | practice |  | In-depth Practicum in Public Health Data |
|  | 18:00∼19:00 | 1 | practice |  | Coordination and Practice Orientation |
| Week 8 | 13:00∼15:00 | 2 | theory | Team Projects | Use Cases for Life-log Data Analytics - Enterprise |
|  | 15:00∼16:00 | 1 | theory |  | Cloud Usage/Analytics Dataset Coaching |
|  | 16:00∼19:00 | 3 | practice |  | Project Team Meetings, Team Project Practice |
| Week 9 | 13:00∼15:00 | 2 | theory | Team Projects | Use Cases for Life-log Data Analytics - Researcher |
|  | 15:00∼16:00 | 1 | practice |  | Cloud Usage/Analytics Dataset Coaching |
|  | 16:00∼19:00 | 3 | practice |  | Project Team Meetings, Team Project Practice |
| Week 10 | 15:00∼17:00 | 2 | theory | Presentation & Graduation Ceremony | Trainee Team Project Presentation & Judging / Expert Q&A |
|  | 17:00∼18:00 | 1 | theory |  | **Graduation Ceremony, Outstanding Project Award** |
| Totality |  | 50 |  | | |
