# Supplementary material for: Impact of Health Informatics Analyst Education on Job Role, Career Transition, and Skill Development: Survey Study
Source: JMIR Med Educ. 2024 Sep 25;10:e54427. doi: 10.2196/54427 (PMC11446175; doi:10.2196/54427)
Supplement: Multimedia Appendix 2 [file mededu-v10-e54427-s002.docx]

**Survey on changes in informatics competency and demand for continuing education among graduates after completing the 「Advanced Health Informatics Analyst」 course**

| **Part I. General Status of Participants** |
| --- |

**A1. What is your gender?**

| Female |
| --- |
| Male |

**A2. How old are you this year? Please enter your date of birth.**

**____ Year ____ Month**

**A3. Where do you currently live?**

※ If you live overseas, you can write the name of your country in the other.

| Seoul |
| --- |
| Daejeon |
| Busan |
| Daegu |
| Incheon |
| Kwangju |
| Ulsan |
| Gyeonggi-do |
| Gangwon Province |
| South Chungcheong Province |
| North Chungcheong Province |
| South Jeolla Province |
| North Jeolla Province |
| Gyeongsangnam-do |
| Gyeongsangbuk-do |
| Sejong Special Self-Governing City |
| Jeju Island |
| Other( ) |

**A4. What is your highest level of education completed?**

| High School Graduation |
| --- |
| Graduation from university |
| Master's Program (during and after the course) |
| Graduated from a master's program |
| Doctoral Program (during and after the course) |
| Graduated from the doctoral program |
| Other( ) |

**A5. Which of the following fields is your Bachelor's Criteria Major?**

| Humanities |
| --- |
| Department of Law |
| Current account series |
| Department of Social Sciences |
| Department of Education |
| Department of Engineering |
| Natural series |
| Department of Medicine |
| Performing Arts |
| Other( ) |

**A5-1. Please describe the specific subjects of your undergraduate major.**

**A6. Which of the following fields is your final academic background (master's degree or higher) based on?**

| Humanities |
| --- |
| Department of Law |
| Current account series |
| Department of Social Sciences |
| Department of Education |
| Department of Engineering |
| Natural series |
| Department of Medicine |
| Performing Arts |
| Other( ) |

**A6-1. Please describe in detail your final academic level (master's degree or higher) and the standard major subjects.**

**A7. Which of the following does your current employment status apply to?**

| **Employment status** | **explanation** |
| --- | --- |
| Commercial wage earners | Even if the contract period is more than one year or the term is not fixed, a person who is hired in accordance with the company's bylaws and is subject to personnel management regulations and receives benefits such as bonuses, allowances, and severance pay. |
| Temporary wage earners | A person whose contract period is more than 1 month but less than 1 year, or a person who is employed because of the need to complete a specific business |
| Daily wage earners | A person whose employment contract period is less than one month, or if the wage is calculated and paid on a daily basis or per diem |
| Employer | A person who has more than one paid employee and manages a business or farm |
| Self-employed | Those who work alone or with unpaid family members without paid employees |
| Unpaid Family Volunteer | Those who do not have a salary but contribute to the income of a self-managed business and work more than 18 hours a week |
| Etc |  |

**A7-1. If you are not employed, please select the situation that is closest to you at this time.**

| Preparing for employment (undergraduate or graduated, etc.) |
| --- |
| Graduate Students (Master's/Ph.D.) |
| On leave of absence |
| Other( ) |

**A8. What is your current specific job? Please select all of them.**

| **Job Type** | **Sub-occupational groups** |
| --- | --- |
| Sales/Service Jobs | Sales, store sales, beauty/dining, security/security-related service jobs, etc. |
| Clerical/Business Management Jobs | Public Enterprises |
|  | Small and medium-sized business |
|  | Large corporations |
| Skilled Workers | Construction/Mining |
|  | Driving/Transporting/Delivery |
|  | Manufacturing/Production Line |
|  | Cleaning management/security |
|  | Other work/skilled occupations (lathe/carpentry, etc.) ( ) |
| Civil servants/quasi-civil servants | The police |
|  | Education/Teacher |
|  | Soldier |
|  | Civil servant |
| Professionals/related workers | Doctor |
|  | Pharmacist |
|  | Legal Profession |
|  | Professor |
|  | Tax/Accounting/Patent Attorney |
|  | Press/Broadcaster |
|  | Researcher |
|  | Nurses and medical professions |
|  | Lecturer |
|  | Other professions ( ) |
| Business owners | Agriculture/fishing/forestry/mining |
|  | Manufacturing/Construction/Transportation |
|  | Wholesale & Retail |
|  | Trade |
|  | Accommodation/Service Industry |
|  | Food & Beverage |
|  | Real estate and leasing |
|  | Other ( ) |
| Student | Bachelor |
|  | Master's Courses |
|  | Phd |
| Etc | Unemployed |
|  | Retirement |
|  | Freelancer |
|  | Other ( ) |

**A9. Which of the following organizations are you currently affiliated with? Please select all of them.**

| Medical institutions |
| --- |
| University/ Graduate School |
| General Enterprises |
| Private Sector Research Institute |
| Public Sector Research Institute |
| Central government and affiliated agencies (Korea Disease Control and Prevention Agency, Ministry of Food and Drug Safety, etc.) |
| Local governments and affiliated organizations |
| Public Institutions |
| Other( ) |
| Independent |

**A10. What were your responsibilities prior to or at the time of completion of the "Advanced Health Informatics Analyst " course? Please select up to 3 in the order of proximity.**

**Rank 1: ___________ Rank 2: ___________ Rank 3: ___________**

| General Manager/ Officer/ CEO |
| --- |
| HR/Finance (HR, Accounting, Fund Management, etc.)/Management Support (General Affairs, Legal Affairs, Office Affairs) |
| Research &D |
| Production/ Manufacturing |
| Hospital care/ check-up |
| Data analysis |
| Computer/IT System Management |
| Sales/Marketing/Planning |
| Service Response/ Desk Response/ Customer Care |
| Education/ Counseling/ Coaching |
| Other ( ) |

**A11. After completing the "Advanced Course in Medical Information Analysis", what are your current duties?Please select up to 3 in the order of proximity .**

**Rank 1: ___________ Rank 2: ___________ Rank 3: ___________**

| General Manager/Officer/Representative |
| --- |
| HR/Finance (HR, Accounting, Fund Management, etc.)/Management Support (General Affairs, Legal Affairs, Office Affairs) |
| Research &D |
| Production/ Manufacturing |
| Hospital care/ check-up |
| Data analysis |
| Computer/IT System Management |
| Sales/Marketing/Planning |
| Service Response/ Desk Response/ Customer Care |
| Education/ Counseling/ Coaching |
| Other( ) |

**A11-1. How long is your current career in the main role?**

**From ________ to ________**

**A12.If you are currently working in informatics/medical informatics, what are you doing? Please select all of them.**

| Patient/clinical data storage management |
| --- |
| User Interface Design |
| Manage data security and privacy |
| Design and maintain clinical or research data registries |
| Clinical Care Support Software/Hardware Maintenance |
| Maintain and improve computer systems in hospitals such as EMR and PACS |
| Data quality and standards management, training |
| Data analysis and optimization |
| Final report and recommendation of all progress related to informatics/medical informatics to decision-makers |
| Informatics/Medical Informatics Student or Staff Training |
| Won and progress national projects in the field of informatics/medical informatics |
| Clinical Data & Software Quality Management |
| Introduction and installation management of medical support software, TOOLS, etc. |
| Advancement of systems for streamlining and automating clinical care processes |
| Technical support for system error correction or troubleshooting |
| Educate users for the use of clinical support systems and software |
| Participation in a multidisciplinary team for the development/advancement of clinical support systems (EMR, PHR, etc.) |
| Conduct real-world data research using clinical data |
| Supporting research using medical information methodologies to solve clinical problems |
| Big data research and development using artificial intelligence and statistical techniques |
| Patient-generated data (lifelog, mobile health) management software development and utilization support |
| Utilization of clinical data, establishment of research support plans, and development of utilization tools |
| Design and conduct multicenter clinical trials |
| Other( ) |
| I do not perform informatics/medical informatics-related work. |

| **Part II. Comparison of Before and After Course Completion** |
| --- |

**B1. What was the purpose or motivation for you to apply for the "Advanced Course in Medical Information Analytics"? Please select all of them.**

| Strengthening or improving one's competence in the performance of one's work (**for the purpose of preparing for one's current job**) |
| --- |
| Acquire knowledge and competencies necessary for collaboration even though it is not your job (**for collaboration** purposes) |
| Acquiring the competencies required for future work (for the **purpose of preparing for future work possibilities**) |
| Your supervisor or institution requires you to complete the course **(on the recommendation of others**) |
| Securing the competencies necessary for employment or changing jobs (for **the purpose of obtaining a certificate and reinforcing your career**) |
| Develop new skills to prepare for an uncertain future (**for self-development purposes**) |
| To obtain information through exchanges with relevant faculty members or students (**for the purpose of obtaining information**) |
| Other( ) |

**B2. To what extent do you think the "Advanced Course for Medical Information Analysis Specialists" has helped (satisfied) you overall?**

| No help (satisfaction) at all  It didn't work | Help (satisfaction)  It didn't work | It's normal | Help (satisfaction)  Was | Very helpful (satisfied)  Was |
| --- | --- | --- | --- | --- |
| 1 | 2 | 3 | 4 | 5 |

**B3. To what extent do you think the "Advanced Course in Medical Information Analysis" has affected your current role?**

| There was no impact at all | There was little impact | It's normal | There was some impact | It was very influential |
| --- | --- | --- | --- | --- |
| 1 | 2 | 3 | 4 | 5 |

**B4. What do you think the "Advanced Medical Information Analysis Specialist" course has had a positive impact on you? Please select all of them.**

| Interacting with people in various fields and achieving the possibility of expanding the field of informatics |
| --- |
| Securing expertise and differentiation in the field of informatics in the workplace |
| Improving the knowledge of individuals related to informatics/medical informatics |
| Gain real-world data experience necessary for academic or job performance |
| Gain practical knowledge and skills that can be applied to the job |
| Securing competencies for employment and job change (obtaining a certificate) |
| Other( ) |
| There was no particular impact |

**B5. Have you had any changes in your work (such as department changes/job changes) since you completed the "Medical Information Analysis Expert Advanced Course"?**

| yes |
| --- |
| No |

**B6. After completing the "Advanced Course in Medical Information Analysis Specialist", have you moved to the field of informatics/medical informatics, or are you willing to change jobs?**

| yes |
| --- |
| No |

**B6-1. If you have changed jobs or intend to change jobs related to informatics/medical informatics, to what extent has the "Advanced Course in Medical Information Analysis Specialist" had an impact?**

| There was no impact at all | There was no impact | It's normal  (50%) | There was an impact | There was a big impact  (100%) |
| --- | --- | --- | --- | --- |
| 1 | 2 | 3 | 4 | 5 |

**B6-2. What was the impact of the "Advanced Course in Medical Information Analysis" on your decision to move to a field related to informatics/medical informatics? Please select all of them.**

| Confirmation of the potential for development in the field of informatics/medical informatics |
| --- |
| Check the degree of alignment of your aptitude with the field of informatics/medical informatics |
| Identify the content and characteristics of work in the field of informatics/medical informatics |
| Improving personal academic skills and acquiring academic qualifications |
| Interest in the field of informatics/medical informatics |
| Interaction with people with interests in the field of medical information |
| Cultivate job performance skills (skills development, data analysis, etc.) |
| Other ( ) |

| **Part III. Curriculum Impact** |
| --- |

**C1. What level of [medical data analysis and processing] do you think you have before and after completing the "Advanced Medical Information Analysis Specialist Course"?**

| **NO** | **category** | Step 0 | Step 1 | Step 2 | Step 3 | Step 4 |
| --- | --- | --- | --- | --- | --- | --- |
|  |  | I had no idea | Listen to the terminology  Understand the concept | You can see and follow the analysis method | Know how to analyze and be able to design | Able to perform expert-level analysis and derivation of results |
| 1 | Before the course is completed | 1 | 2 | 3 | 4 | 5 |
| 2 | After completing the course | 1 | 2 | 3 | 4 | 5 |

**C2. What skills and skills do you have improved in informatics/medical informatics before and after completing the "Advanced Medical Information Analysis Specialist Course"? Please select all of them.**

| Understanding the healthcare system and the healthcare delivery process |
| --- |
| Data analysis using programming languages |
| Healthcare Data Utilization Research Methodology and Study Design |
| Developing and leveraging digital technologies to develop and utilize healthcare services |
| Understand the characteristics of multidimensional medical data such as imaging, public health, and genomic |
| Other( ) |

**C3. If any of the following tasks are currently being performed, please select all of them.**

| According to the analysis goal, we plan the analysis method of big data |
| --- |
| Collect big data resources for analysis |
| Preprocess the collected data |
| We use processing technologies such as collection, storage, extraction, and integration of big data to build an optimal analysis system |
| Process the data and create an analysis report that applies various modeling techniques |
| Visualize the results to help you understand the main analysis results |
| Identify new technologies, trends, and trends related to big data |
| Other( ) |
| NA |

**C4. How has your interest in informatics/medical informatics changed since you completed the "Advanced Course in Medical Information Analysis"?**

| Interest has declined significantly | Decreased | No change | Increased | Interest has increased significantly |
| --- | --- | --- | --- | --- |
| 1 | 2 | 3 | 4 | 5 |

**C5. After completing the "Advanced Course in Medical Information Analysis", how did your fear of using informatics/medical informatics change?**

| Fear has increased significantly | Fear decreased slightly. | No change | Fear decreased slightly | Fear  It was very reduced |
| --- | --- | --- | --- | --- |
| 1 | 2 | 3 | 4 | 5 |

**C6. How has your level of participation in activities related to informatics/medical informatics changed since you completed the "Advanced Medical Information Analysis Specialist Course"?**

| Participation in activities  significantly reduced | Decreased | No change | Increased | Participation in activities  Significantly increased |
| --- | --- | --- | --- | --- |
| 1 | 2 | 3 | 4 | 5 |

**C7. After completing the "Advanced Course for Medical Information Analysis Specialists", what has increased your interest or activity in informatics/medical informatics? Please select all the items that have increased weight.**

| Informatics/Medical Informatics related courses (online/offline) and plan to take courses |
| --- |
| Obtain and plan to obtain a degree related to informatics/medical informatics |
| Utilizing informatics/medical informatics methods in actual work |
| Informatics/medical informatics-related community activities (study groups, Internet café membership, etc.) |
| Search for information related to informatics/medical informatics (papers, magazines, articles, etc.) |
| Attending conferences related to informatics/medical informatics |
| Publication of academic research related to informatics/medical informatics |
| Other( ) |
| NA |

**C8. If you have found it difficult to engage in informatics/medical informatics after completing the "Advanced Course in Medical Information Analysis Specialist", what is the reason? Please select all of them.**

| By achieving other objectives set out by the individual. |
| --- |
| It's not what I expected |
| Due to the difficulty of the curriculum (too difficult.) |
| There is a gap with the actual work. |
| Recently, I have been more interested in other fields |
| Because it doesn't suit my aptitude |
| There are no further continuing education opportunities after the enrichment course. |
| Because there is no corporate demand for human resources in related fields. |
| The salary of the personnel who perform work in the related field is low or the working environment is not good. |
| Other ( ) |
| NA |

| **Part IV. Continuing Education Needs** |
| --- |

**D1. After completing the "Advanced Course for Medical Information Analysis Specialists", how much do you think you need a linkage or reinforcement training course?**

| Not necessary at all | It is not necessary | It's normal | need | It is very necessary |
| --- | --- | --- | --- | --- |
| 1 | 2 | 3 | 4 | 5 |

**D1-1. If there is a linkage or reinforcement training course related to the "Advanced Course for Medical Information Analysis Specialists", what are your current needs? Select up to 3 in the order you need .**

**Rank 1: ___________ Rank 2: ___________ Rank 3: ___________**

| Practice building IT infrastructure for big data analysis |
| --- |
| Know-how to design and build CDSS using medical artificial intelligence |
| Development of PHR application based on lifelog data |
| Conducting research on the linkage of public data and medical data |
| Experts in data preprocessing and standardization for the use of medical big data |
| Development of deep learning models using medical signal data |
| Development and utilization of medical form data natural language processing technology |
| Medical data quality management and evaluation technology |
| Medical Artificial Intelligence and Big Data Law and Ethics Training |
| Practical and patent know-how related to start-up founding using medical data |
| Medical data information protection and security technology |
| Other( ) |
| Not required |

**D1-1-1. How do you want the training you need to be delivered? Please select up to 3 methods in the order you want.**

**Rank 1: ___________ Rank 2: ___________ Rank 3: ___________**

| Offline training | Theoretical lectures |
| --- | --- |
|  | practice |
|  | Team Projects |
|  | Workshop |
|  | Topic Discussion |
| Online Training | Theoretical lectures |
|  | practice |
|  | Team Projects |
|  | Topic Discussion |
| Training linked to companies and medical institutions | Internship |
|  | Participation in collaborative research |
| Other( ) | |

**D2. To what extent do you think there is a need for "professional training" related to informatics/medical informatics?**

| Not necessary at all | It is not necessary | It's normal | need | It is very necessary |
| --- | --- | --- | --- | --- |
| 1 | 2 | 3 | 4 | 5 |

**D3. Based on your current organization, how do you predict the staffing needs related to informatics/medical informatics will change in the future?**

| It will be greatly reduced. | It will decrease somewhat. | There will be no change. | It will increase somewhat. | It will increase significantly. |
| --- | --- | --- | --- | --- |
| 1 | 2 | 3 | 4 | 5 |

**D4. Lastly, if you have any improvements or suggestions related to the "Medical Information Analysis Expert Intensive Course", please be specific.**

|  |
| --- |
