# Supplementary figures and images for: Impact of Health Informatics Analyst Education on Job Role, Career Transition, and Skill Development: Survey Study
Source: JMIR Med Educ. 2024 Sep 25;10:e54427. doi: 10.2196/54427 (PMC11446175; doi:10.2196/54427)

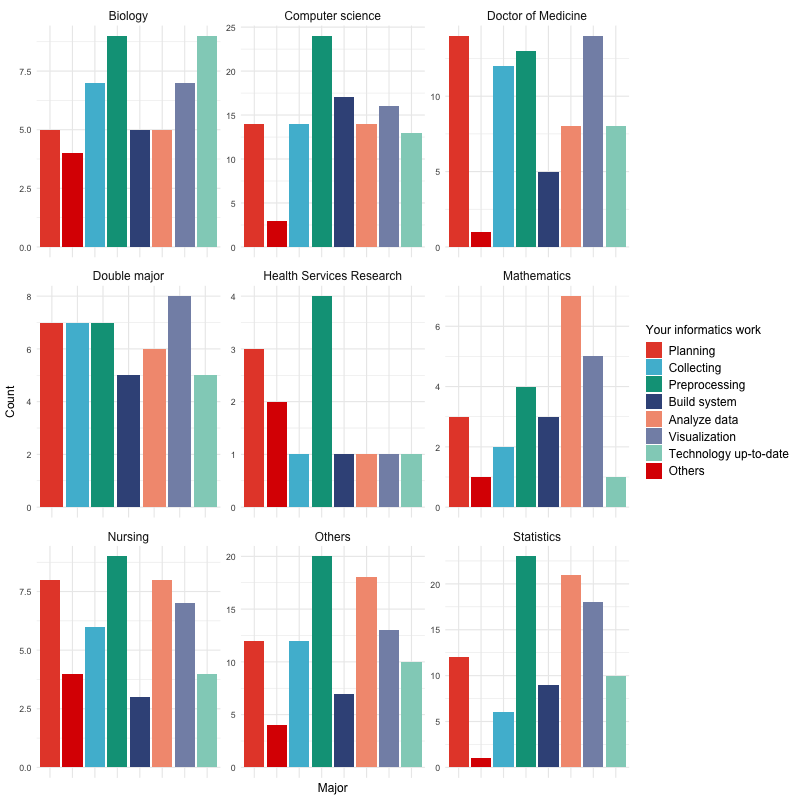

Supplement: Multimedia Appendix 3 [file mededu-v10-e54427-s003.png]
